# The Views and Needs of People With Parkinson Disease Regarding Wearable Devices for Disease Monitoring: Mixed Methods Exploration

**DOI:** 10.2196/27418

**Published:** 2022-01-06

**Authors:** Lorna Kenny, Kevin Moore, Clíona O' Riordan, Siobhan Fox, John Barton, Salvatore Tedesco, Marco Sica, Colum Crowe, Antti Alamäki, Joan Condell, Anna Nordström, Suzanne Timmons

**Affiliations:** 1 Centre for Gerontology and Rehabilitation School of Medicine University College Cork Cork Ireland; 2 Tyndall National Institute University College Cork Cork Ireland; 3 Research, Development and Innovation Activities & Physiotherapy Education Karelia University of Applied Sciences Joensuu Finland; 4 School of Computing, Engineering and Intelligent Systems Faculty of Computing, Engineering and the Built Environment Ulster University Magee United Kingdom; 5 Department of Public Health and Clinical Medicine Umeå University Umeå Sweden

**Keywords:** Parkinson disease, wearable devices, technology, mixed method, focus group, survey, mobile phone

## Abstract

**Background:**

Wearable devices can diagnose, monitor, and manage neurological disorders such as Parkinson disease. With a growing number of wearable devices, it is no longer a case of whether a wearable device can measure Parkinson disease motor symptoms, but rather which features suit the user. Concurrent with continued device development, it is important to generate insights on the nuanced needs of the user in the modern era of wearable device capabilities.

**Objective:**

This study aims to understand the views and needs of people with Parkinson disease regarding wearable devices for disease monitoring and management.

**Methods:**

This study used a mixed method parallel design, wherein survey and focus groups were concurrently conducted with people living with Parkinson disease in Munster, Ireland. Surveys and focus group schedules were developed with input from people with Parkinson disease. The survey included questions about technology use, wearable device knowledge, and Likert items about potential device features and capabilities. The focus group participants were purposively sampled for variation in age (all were aged >50 years) and sex. The discussions concerned user priorities, perceived benefits of wearable devices, and preferred features. Simple descriptive statistics represented the survey data. The focus groups analyzed common themes using a qualitative thematic approach. The survey and focus group analyses occurred separately, and results were evaluated using a narrative approach.

**Results:**

Overall, 32 surveys were completed by individuals with Parkinson disease. Four semistructured focus groups were held with 24 people with Parkinson disease. Overall, the participants were positive about wearable devices and their perceived benefits in the management of symptoms, especially those of motor dexterity. Wearable devices should demonstrate clinical usefulness and be user-friendly and comfortable. Participants tended to see wearable devices mainly in providing data for health care professionals rather than providing feedback for themselves, although this was also important. Barriers to use included poor hand function, average technology confidence, and potential costs. It was felt that wearable device design that considered the user would ensure better compliance and adoption.

**Conclusions:**

Wearable devices that allow remote monitoring and assessment could improve health care access for patients living remotely or are unable to travel. COVID-19 has increased the use of remotely delivered health care; therefore, future integration of technology with health care will be crucial. Wearable device designers should be aware of the variability in Parkinson disease symptoms and the unique needs of users. Special consideration should be given to Parkinson disease–related health barriers and the users’ confidence with technology. In this context, a user-centered design approach that includes people with Parkinson disease in the design of technology will likely be rewarded with improved user engagement and the adoption of and compliance with wearable devices, potentially leading to more accurate disease management, including self-management.

## Introduction

### Background

Parkinson disease is a progressive, chronic neurodegenerative disorder. The disease is characterized by motor symptoms including tremor, rigidity, bradykinesia, dyskinesia, and nonmotor symptoms such as cognitive impairment, fatigue, and pain [1,2]. Globally, Parkinson disease is the second most common neurodegenerative disease, affecting >6 million people worldwide [3], with an estimated prevalence of 1% in people aged ≥60 years and 2% in people aged ≥80 years [4]. As the median age increases in many countries, so does the prevalence of Parkinson disease [3]. This increased prevalence carries a personal and societal burden, with an estimated cost to society of US $22,800 per patient, per year [5].

Owing to the heterogeneity and complexity of Parkinson disease features, its clinical assessment may be challenging, relying on sporadic, subjective clinician assessment and self-evaluation of symptoms by patients [6]. Symptom diaries can be onerous to complete, whereas infrequent clinical examinations offer limited insight into the day-to-day symptom fluctuations [7]. In contrast, wearable devices can collect continuous, accurate, and objective data over a prolonged period. They quantify motor fluctuations, collect key data during critical events (eg, whether dramatic motor decline is due to bradykinesia or hypotension), and continuously monitor motor symptoms [8]. Wearable devices have been used to identify and quantify bradykinesia [9], tremor [10], postural sway [11], motor fluctuations [12], and dyskinesia [13]. They have also been used to measure gait [14], sleep disorders [15], falls [16], and physical activity levels [17]. On the basis of the deliberations of the International Parkinson and Movement Disorders Society Task Force on Technology, Espay et al [8] summarized how technology-based objective measures may decrease clinical visits, engage patients in their own care, and close the loop between clinicians and their patients.

Previous research has identified the general research priorities for people with Parkinson disease, including improving motor and nonmotor symptoms, mental health issues, medication side effects, interventions specific to Parkinson disease, better monitoring systems, and quality health care [18-20]. People with Parkinson disease have already expressed their desire for wearable devices to capture data on a range of symptoms and lifestyle factors. They want the technology to capture data on bradykinesia, tremor, balance, gait, sleep, and cognition [21]. Design interface, usability, and accuracy are important aspects, and people with Parkinson disease want an unobtrusive design [22-25]. They want to understand which data are collected before, during, and after monitoring. In addition, they want to be assured that the information gathered is worthwhile and clinically relevant to them and that it captures Parkinson disease symptom fluctuations [25]. Additional desirable features include specialized Parkinson disease functions, such as real-time detection of motor fluctuations and medication prompts [26]. The importance of interactive communication and feedback between the devices, patients, and health care professionals is evident [25]. Although many older adults embrace technology, people aged >65 years generally use fewer new technologies and use them less frequently [27]. Older adults use technology for emailing, web-based searching, and web-based shopping, but less so for connected health [28]. Older adults with ability limitations appear to use technology even less frequently [29]. Similarly, poor confidence in handling new technology has been highlighted as an area of concern for people with Parkinson disease; therefore, wearable devices should be easy to learn and use [23].

### Objectives

The aforementioned feedback was derived from a variety of study designs. To our knowledge, much of the previous research in this area has used surveys to solicit feedback from people with Parkinson on specific devices only [22,24,30] or included the views of people with Parkinson disease alongside other populations such as people with epilepsy [25]. With a growing number of wearable devices on the market, it is no longer simply a case of whether a wearable device can measure Parkinson disease motor symptoms but rather of which features best suit the user’s needs. Previous research has outlined the motor symptoms that a wearable device should measure [21], and the latest devices can accurately measure these [31]. Thus, concurrent with continued device development, we must generate insights on the nuanced needs of the user in the modern era of wearable device capabilities.

As part of a larger European study on wearable devices for remote rehabilitation of older people (SENDOC [Smart Sensor Devices for Rehabilitation and Connected Health]), we aim to explore, using a mixed method approach, the views and needs of people with Parkinson disease who are aged ≥50 years, regarding wearable devices for monitoring, treatment decisions, and care-planning.

## Methods

### Study Design

This study used a mixed method parallel design, wherein surveys and focus groups were concurrently conducted with people with Parkinson disease in Munster, Ireland. There have been calls in the technological literature to use a mixed method approach to produce meaningful understanding when studying complex contexts [32]. This approach is quite novel in Parkinson disease wearable device research, where previous mixed method studies have mainly focused on disparate populations or on specific devices only. A mixed method approach combines the advantages of quantitative and qualitative methodologies, enabling the collection of rich data that reflect the participants’ perspectives and ensures that the study findings are rooted in their experience [33]. Qualitative research methods are well suited to examine user needs and may offer explanations for unexpected or anomalous findings in quantitative data [34] or uncover usability barriers that quantitative approaches often miss.

Members of a local branch of the Parkinson’s Association of Ireland (PAI) formed part of the research team and guided the study design and advised on patient recruitment. These advisers consisted of 2 people with Parkinson disease and a caregiver of a person with Parkinson disease. The advisory group helped identify and prioritize the research questions and shaped the data collection tools. They also assisted in the recruitment of participants for the focus groups and the distributed surveys. Upon completion of the research, the advisers guided the researchers on how best to disseminate the results to people affected by Parkinson disease.

### Participants

The inclusion criteria for the survey and focus groups were as follows: age ≥50 years and a diagnosis of Parkinson disease. The focus group participants were selected using a criterion theoretical sampling strategy to satisfy the following criteria: inclusion of different age categories (50-60 years, 61-70 years, 71-80 years, >80 years) and the inclusion of men and women.

Participants were excluded if they had significant communication deficits. Ethical approval was obtained from the Clinical Research Ethics Committee of the University College, Cork.

Participants were recruited through local branches of PAI, a not-for-profit advocacy and support group, with researchers or PAI research volunteers attending local branch meetings to inform the attendees (people with Parkinson disease and their families). Those expressing interest were given an information sheet about the study, and a copy of the survey which was to be returned later to the PAI branch if desired.

### Survey

The survey consisted of structured questions in 2 parts. Part A included 8 items including gender, age, experience with technology, and knowledge of wearable devices. Part B contained 18 Likert items that probed the importance of certain wearable device features across 4 thematic categories: *wearability*, *user interface*, *wearer feedback*, and *clinical accuracy*. This survey was closely based on the survey by Bergmann and McGregor [35], with 5 items unchanged, 8 with minor wording changes to improve understanding, and 5 new or substituted items based on the advisory group’s feedback. These included the device being rechargeable, sending alerts, and giving the user ownership of their own health care (see Multimedia Appendix 1 for the full survey, mapped to the original Bergmann survey). The 10-point Likert response format from Bergmann’s survey was converted to a 5-point Likert scale to make it easier for older participants to complete. Items were thus presented as *strongly agree*, *agree*, *neutral*, *disagree*, and *strongly disagree*. By completing the anonymous survey and returning it to the PAI branch, participants gave their consent to participate (signed consent would have removed anonymity).

### Focus Groups

Focus groups took place in local community spaces throughout Munster, Ireland. Written informed consent was obtained from all the patients in advance.

The semistructured topic guide questions explored the priorities of people with Parkinson disease in everyday life; the usefulness of wearable devices, their perceived benefits, and the barriers to use; and the usability and important design features.

Initial drafts of the topic guide were based on existing literature and the purpose of the study. These were iteratively reviewed by the advisory group and the researchers together in terms of the content, focus, and relevance to people with Parkinson disease (see Multimedia Appendix 2 for the final focus group schedule). The focus group guide was used to ensure that all topics were covered, but the groups were informal and interactive to obtain as many insights as possible, lasting from 50 to 70 minutes. During the focus group, participants were shown examples of a wearable smart watch and a glove device. However, the participants did not try on or take these devices to their homes. They were used as tools to demonstrate examples of wearable technology to encourage discussion.

### Data Analysis

Simple descriptive statistics were used to represent the data for the Likert items in part B of the survey. Given that the participants rated all statements positively (no one selected disagree or strongly disagree for any Likert items), the data have been presented as the ratio between the categories of neutral, agree, and strongly agree responses. A higher proportion of strongly agree responses for an item indicated its importance to the participants. No statistical tests were conducted because of the positive skew of the Likert statements and the invalidated status of this survey tool. In part A of the survey, Fisher exact test and the Wilcoxon signed rank test were used to test the difference between men and women for each survey response.

The focus groups were audiotaped and transcribed. Transcripts were analyzed for common themes using a qualitative thematic approach [36]. A lead researcher (highly familiar with this approach) used open coding to collect data from the participants’ views. The coding scheme combined an inductive and deductive process, where codes were assigned as appropriate; however, the researcher also deductively decided how these codes fitted with the categories of the survey (ie, wearability, user interface, wearer feedback, and clinical accuracy). Transcript data were broken down into discrete excerpts, labeled, and described, with the coding remaining tentative and subject to change as it continued in subsequent rounds. A second researcher (SF) reviewed a sample of the transcripts and codes, and both researchers established an agreement on the final codes. Codes were then grouped into provisional subthemes and themes, and key phrases were later assigned according to their content. Themes were designated on the foundation that they echoed the patterns of participant responses in the transcript data and were significant to our research question [36], that is, *what are the views and needs of people with Parkinson disease regarding wearable devices for monitoring, treatment decisions, and care-planning?*

The survey and focus groups were conducted concurrently and analyzed separately. The results were *integrated through narrative*, where the qualitative and quantitative results were described and woven together on a theme-by-theme basis [37]. Findings from each type of data confirmed the results of the other type and provided similar conclusions, which increased their reliability [38]. This representation procedure ensured coherent data integration and allowed a fuller depiction of the views and needs of people with Parkinson disease regarding wearable devices [39].

## Results

### Participants

The people with Parkinson who attended the meetings, and hence participated in the surveys and focus groups, were typically at Hoehn and Yahr stage 1 to 3 (informal impression from a Parkinson disease expert, ST, from attendance at a meeting). There were 32 surveys completed by people with Parkinson disease. Of the respondents, 56% (18/32) were male and 44% (14/32) were female, ranging in age from 50 to 83 years, with a median age of 68 years. The response rate was not captured because the surveys were disseminated by a PAI member volunteer who distributed surveys to group members who had attended meetings over a period of 6 months. The PAI volunteer reported that, to their recollection, every dyad who attended the meetings completed the survey, with no refusals, and that most people with Parkinson disease completed the survey alone and some completed it with their family members’ support.

In part A of the survey, the results of the statistical tests showed no significant differences between males and females for any of the responses (*P*>.60). The results for part A have been presented in Table 1. For part B of the survey, statistical tests were not performed. The results have been presented in Table 2 as a percentage selection, grouped by category.

Overall, 4 semistructured focus groups were held with 24 people with Parkinson disease, with 4 to 7 participants in each group. In all, 14 men and 10 women participated, ranging in age from 53 to 84 years with a median age of 70.5 years.

**Table 1:** Technology use among participants.

		All (N=32)	Female (n=14)	Male (n=18)
Age (years), median (range)		68 (46-83)	68.5 (46-78)	66.5 (53-83)
**Current technology use, n (%)**				
	Smartphone	18 (58)	8 (57)	10 (59)
	Games console	2 (6)	1 (7)	1 (6)
	Desktop computer	25 (81)	11 (79)	14 (82)
	Any of the above	27 (87)	12 (86)	15 (83)
	Other technology	2 (6)	2 (14)	0 (0)
	Missing data	1 (3)	0 (0)	1 (6)
**Self-rating of technology skills, n (%)**				
	No skills	4 (13)	2 (14)	2 (11)
	Poor skills	3 (9)	1 (7)	2 (11)
	Average skills	10 (31)	5 (36)	5 (28)
	Good skills	14 (44)	5 (36)	9 (50)
	Excellent skills	1 (3)	1 (7)	0 (0)
	Missing data	0 (0)	0 (0)	0 (0)
**Frequency of technology use, n (%)**				
	Every day	24 (77)	11 (79)	13 (76)
	Most days	3 (10)	1 (7)	2 (12)
	Every week	0 (0)	0 (0)	0 (0)
	Rarely	3 (10)	2 (14)	1 (6)
	Missing data	1 (3)	0 (0)	1 (6)
**Have heard of wearable devices, n (%)**				
	Yes	20 (65)	7 (50)	13 (76)
	Missing data	1 (3)	0 (0)	1 (6)
**Have used a wearable device, n (%)**				
	Yes	11 (35)	3 (21)	8 (47)
	Missing data	1 (3)	0 (0)	1 (6)
**Currently using store-bought wearable device, n (%)**				
	Yes	2 (6)	1 (7)	1 (6)
	Missing data	1 (3)	0 (0)	1 (6)

**Table 2:** Responses to Likert statements about wearable device features and capabilities^a^.

Statement			Strongly agree, n (%)		Agree, n (%)		Neutral, n (%)		Missing, n (%)
**Wearability of a medical sensing device (n=180)**			116 (64)		58 (33)		6 (3)		N/A^b^
	Should be comfortable to wear^c^	17 (53)		11 (34)		2 (6)		2 (6)	
	Should be compact (light and small)^c^	17 (53)		12 (38)		1 (3)		2 (6)	
	Should be discrete^c^	17 (53)		12 (38)		1 (3)		2 (6)	
	Should be easy to attach to the body^c^	22 (69)		8 (25)		0 (0)		2 (6)	
	Should not affect your normal daily routine^c^	21 (66)		8 (25)		1 (3)		2 (6)	
	Should not detach accidently^c^	22 (69)		7 (22)		1 (3)		2 (6)	
**User interface (n=90)**			60 (67)		30 (33)		0 (0)		N/A
	Should be rechargeable^c^	19 (59)		11 (34)		0 (0)		2 (6)	
	Should be simple to operate (and maintain)^c^	20 (63)		10 (31)		0 (0)		2 (6)	
	Should be accompanied by clear and readable instructions for use^c^	21 (66)		9 (28)		0 (0)		2 (6)	
**Wearer feedback (n=90)**			52 (58)		37 (40)		1 (1)		N/A
	Should give instant feedback to you	17 (53)		12 (38)		1 (3)		2 (6)	
	Should send alerts to the user	20 (63)		10 (31)		0 (0)		2 (6)	
	Should provide you with alerts, that is, performance versus target (eg, step count)	15 (45)		15 (45)		0 (0)		3 (9)	
**Clinical accuracy (n=175)**			143 (82)		32 (18)		0 (0)		N/A
	Should be reliable	24 (75)		6 (19)		0 (0)		2 (6)	
	Should increase the accuracy of current clinical assessment	24 (75)		5 (16)		0 (0)		3 (9)	
	Should reduce your requirement to travel for clinical assessment	23 (72)		6 (19)		0 (0)		3 (9)	
	Should form part of your clinical assessment	24 (75)		5 (16)		0 (0)		3 (9)	
	Should give you a sense of ownership of your own health care	24 (75)		5 (16)		0 (0)		3 (9)	
	Should work alongside your medical care team, instead of replacing them	24 (75)		5 (16)		0 (0)		3 (9)	

^a^No participant selected *Disagree* or *Strongly Disagree* for any Likert item.

^b^N/A: not applicable.

^c^n=30.

### Survey and Focus Group Results

The first major theme below was not addressed in the survey. For all other themes, the survey data have been first presented, followed by the relevant subthemes from the focus group.

#### Living With Parkinson Disease

At the beginning of the focus group, participants shared what they felt were important aspects of daily life for people with Parkinson disease.* *The participants valued their independence and wanted to remain independent for as long as possible:

Independence. That is my goal. Hits it on the head. I'd love to be independent. 

It's trying to live as normal a life, as close to it as possible, and adapting where necessary and that's the other thing, you're having to develop a whole set of different skills. 

Participants discussed a range of different challenges they faced with Parkinson disease, including motor function and tremor. They described their problems with bradykinesia and the resulting frustration:

The slowness I find, I gets angry now and frustrated. It takes maybe half an hour to do what I can do in five minutes.

It takes me a long time, you know. The dogs aged another dog year by the time I'm finished. It's that type of thing. That's the slowing down which I've struggled with myself.

Falls, and the risk of falling was also a common concern expressed by the participants: “People with Parkinson’s tend to get falls, quite a lot of falls, and means that basically [falls] take them out of circulation for months.” Participants described how *normal day to day things and continuing the routine are essential* in managing the condition effectively, especially for medication regimes, daily activities, exercise, and sleep. The benefits of exercise on Parkinson disease symptoms were viewed as an important part of maintaining health, and participants shared personal experiences of how various exercise regimes helped improve both motor and nonmotor symptoms. The group felt that *exercise really is essential* and physical activity should be encouraged for people with Parkinson disease:

I have to maintain a certain level of activity to keep going as in walking or some form of exercise, and even this past week now I haven't been as consistent or as regular and I find that I'm stiffening a little bit first thing in the morning...I have to do it because otherwise I won’t be able to keep going or it'll slow me down that bit more.

Sleep was suggested as another vital element for overall health and well-being, but obtaining adequate rest was often challenging for the participants, as many experienced difficulty sleeping: “If I’ve a bad night, I’d be completely thrown all the next day.” The positive impact of good quality, accessible, and timely health care was discussed. The participants highlighted the importance of regular access to quality health care to alleviate Parkinson disease symptom deterioration:

It’s really important to me to see my GP and consultant regularly. My GP is great cause she’s always linking in with the consultant to make sure we’re on the right track with medication and treatment. As we all know it’s key to get the right support.

However, many experienced difficulties in accessing appropriate specialized Parkinson disease health care in Ireland, including long waiting times to see a consultant:

It's the lack of the go to person, this lack of the proper support nurse. You have your GP who doesn't seem to understand it that well. Or that he refers you onto your consultant who is not easily accessible. There's that gap in the middle. 

You could be waiting months for an appointment with a consultant and it’s just not good enough.

It was also felt that geographic barriers existed within certain areas:

We're in a very black hole in this area. In fact, XX generally is very poorly serviced...Consultants seem to be drifting away, retiring or going to the private sector and oh you can get good care here, but it'll cost you. That's what you've got here. Generally, there is very little and what there is a long time coming, you know.

#### Technology Use

The technology use of survey participants has been summarized in Table 1. Most participants used at least one form of technology daily and rated their skills as average or good. However, although many participants had heard of wearable devices, only a fraction were currently using a purchased wearable device.

The focus groups mirrored this, with technology use most commonly being in the form of desktop computers and smartphones. People used technology to set personal alarms for medication reminders:

The phone is handy. I set alarms. In a half an hour, this phone will go and I’ll know to take the tablets. 

I use it [technology] for medication. I have alarms set to take medication and it reminds me then when to take the next dose. 

Two participants in the focus group used medical devices such as medication-administration pumps: “I wasn't [independent] up to five weeks ago but I am now again, with the help of a machine, a pump.” In addition to mechanical aids in the kitchen, such as a *kettle cradle* or adapted cutlery, a few participants used technology aids for daily living, such as for writing or typing difficulties: “I do and I find now I'm trying to make use of voice to text apps and voice apps from documents.” Such technologies were considered to have improved users’ lives, and it was felt that *health technology can be good for protecting the well-being of older people* and *making just your everyday things a little bit better and a little more comfortable.*

Generally, participants saw a role for technology; however, they felt it should be supplementary to appropriate health care delivery, not a replacement for it:

Personally I think quality primary services are the first things to get right, then you look at what you can do then afterwards with technology. That's what I think anyway. 

Similar to the survey respondents, participants from the focus groups were familiar with wearable monitoring technology; however, no participant was currently using a purchased wearable device. Nevertheless, the participants identified that wearable devices may be conceivably beneficial and acceptable for people with Parkinson disease: “There's no sense that in the main those with Parkinson's may be technophobes that they may not like using that technology cause many do use it often and competently.” Participants suggested that wearable devices may provide the opportunity to measure and monitor the individual variability of motor and nonmotor symptoms of Parkinson disease and could provide opportunities for collecting clinical parameters such as medication, balance, tremor, gait, sleep, and exercise:

I'm all for technology and anything that may aid me to walk. If there was a device that could help do that or if there's any kind of device that through monitoring could help me improve, that would really help me.

#### Clinical Usefulness

Clinical usefulness was the highest rated thematic category in the survey (Table 2). People with Parkinson disease felt strongly that a device should increase clinical accuracy, reduce travel, and integrate as part of their care team. In the focus groups, participants emphasized that technology should objectively monitor their symptoms, and it was deemed beneficial if this information could be relayed to their medical care team:

Something that could monitor your symptoms perhaps and even if there was a way that this could be sent to your doctor or whoever. That would be helpful.

It can be hard to describe symptoms to your consultant, so something that offers an independent evaluation of how you are would be great.

A wearable device that captures Parkinson disease data could provide richer health information to clinicians, which may aid in the improved assessment of Parkinson disease:

We try and write what's happening in advance because the clinicians time is precious, your cash is precious cause you're paying him a fee to assess you, and if you don’t have the information he can't judge.

The capturing of individual health parameters would eliminate the need for diary-based recording of symptoms, which was believed to be problematic at times:

But I think if you got an overall, if you got to know yourself. I write things down at night, but at night I'm wrecked. We all are, and you forget half of it. It would be your individual information. 

Supporting medication dosing or timing was highlighted as a main area of focus:

I think even listening to the accounts here this morning that patients are experiencing times when they have inadequate medication and when they're over medicated. And if the [device] can level that out in some way, that would be ideal. 

Some mechanism of determining what your dopamine level is in your brain. As regards telling you what should you be taking; maybe an inhaler that you could use. Or something along those lines that you could have a kind of monitoring of your dopamine level and an appropriate response process to it. Because sometimes, when I get a bit of heaviness in my head, I think is I because I haven't taken enough medicine or because I've taken too much. Like right now, I've taken enough, but sometimes I couldn't actually say whether or which.

Although it was important for health care teams to have access to the data, people also wanted a sense of ownership over their own health care:

Knowledge is power. The more you know the more you can meet it and anticipate it. 

One focus group included a discussion on whether medical professionals would like technology that could alter the dosage of medicines they prescribe and that this may require a change in attitude from some health professionals:

But the way medicine is controlled in the sense the pharmacist gives out what the prescription is. If you were to have medication that you could adjust accordingly that requires a completely different mind-set and I don’t know would the professionals go for it. 

#### Wearability and User Interface

Survey respondents highly rated a small, easy-to-use device that would not interfere with their routine; they responded strongly agree, with 64.4% (116/180) and 67% (60/90) overall score, to the categories for wearability and user interface, respectively (Table 2).

The wearability and usability of wearable devices for Parkinson disease similarly emerged in the focus groups, and it was felt that wearable device design should consider the user needs to ensure compliance and adoption. Although the size and weight of the device were important, the esthetics of the device appeared less significant: “I don’t care how it looks but if something is hard to use for a person with PD then they’re not going to use it.” Participants felt that wearable device design should consider the complexity of Parkinson disease symptoms, especially motor dexterity: “I don't know if that technology can be adapted for people with Parkinson’s who have these issues with making precise movements.” Participants also discussed the importance of ease of use, device weight, and a design which reflected the needs of people with Parkinson disease:

Now it would matter if it was easy to take on and off, that sort of thing, or that it wasn’t heavy or getting in the way.

It needs to be developed...to have things that are liveable. You don't want it bulky, you don’t want anything that's too small. It's got to be suitable for a person with Parkinson’s.

In addition, a participant described that given Parkinson disease variability, “technology for us needs to be multifactorial.”

Participants felt that although most participants felt comfortable with technology, some may feel intimidated:

There are some very confident people around, but there is clearly a large cohort of people who feel excluded by technology. They find it a bit impenetrable.

Designs should make allowances for a person’s possible lack of knowledge or exposure to a specific technology. It was discussed how some would need general guidance to feel more comfortable, which would also enable greater user adoption of the device:

Another thing for me is having someone help me to understand technology or whatever the technology is. Like a step by step, on how to use it properly. That way we’d all get a lot more out of it.

Another possible barrier was the cost of technology; wearable devices should not be expensive, as this may exclude some people with Parkinson disease: “The cost of something would be important cause that might mean some people can get access but others couldn’t.”

#### Feedback From the Device

Wearer feedback was the least highly rated category in the survey. Most participants selected strongly agree 58% (52/90) of the time with statements about the device providing alerts and instant feedback (Table 2).

The importance of wearable device feedback similarly emerged in the focus groups, where one participant detailed how they wanted to receive as much feedback as possible from a device that captured their data: “...in relation to everything really, to the dyskinesia, to the memory, to whether I’m upright or not.” This would not only be useful for the user but would also be valuable for their health care team: “I would see improvement in the trend or if I was getting worse and my doctor would also see that.” Motor symptom fluctuations resulting from medication wearing off was seen as an important area where feedback from a wearable device would be useful: “There could be things that could help to let you know when something is escalating, shuffle at a particular time and you could get information on that to feedback in terms of stuff wearing off.” In addition, prevention and detection of falls and feedback on balance and coordination were highlighted:

I find with balance that's the big problem with me. This sounds funny now but I'd love something to tell me what way I'm facing, you know. My head is sideways or up or down and I kind of go sideways when I'm walking, you know. If there's some kind of thing that would tell you that whether you're walking straight or not. Some kind of feedback mechanism.

Monitoring and feedback of blood pressure was also a desirable feature: “The blood pressure yes because once the blood pressure goes down that's when you could fall.” Participants suggested that data collected could be used to activate a response or intervention. For example, a device that could activate an alarm in case of a fall and automatically call for assistance would be advantageous: “There are certain ways it could maybe detect a fall and send you an alarm and call somebody then you know attached to the patient.” The automatic recording of data from a wearable device (as opposed to a user-triggered device) was considered important: “Maybe everybody might not be so alert that they could monitor everything so that the automatic option would be good.” However, people would also like to have the option to interact with or input specific information to the wearable device, if desired:

I think there could be a stage 1 and stage 2, I suppose. Stage 1 could just be the monitoring and stage 2 would be manipulate it.

The ability of a wearable device to alert the person to nonmotor symptoms, such as mood, stress, and sleep, was stated as important:

Mood, if you're getting stressed and you don't realise it. Suddenly you're having a panic attack. Kind of like a warning sign that you might be. I wonder can technology look at that kind of stuff, mood, sleep and so on. 

People were especially interested in being able to know how their data compared with the data of other people with Parkinson disease and the potential solidarity that it could bring through knowing they were not alone in a certain symptom:

There are times where I'd like to know where I'm at on the scale, to see the measurement of where I am.

So if you go to the nurse specialist...and you think that you've just got something that's pertinent to you, and only you feel it, and then she says oh yeah but somebody else had it. Because she maybe has a catalogue live and suddenly you don’t feel so isolated and then you can compare it maybe...rather than you just trying plough a lonely furrow.

## Discussion

### Principal Findings

This study explored the views of people with Parkinson disease regarding wearable devices for the management of Parkinson disease. The participants highlighted the challenges of living with a progressive disease, difficulties accessing quality health care, difficulty maintaining independence, and the importance of exercise and sleep. They discussed ways in which wearable devices could benefit their lives and the priorities for future research.

This study suggests that wearing a device is both feasible and acceptable to people with Parkinson disease, as most participants frequently used technology and were receptive of and familiar with health technology. Some participants already used medication reminders, medication-administration pumps, and fall-detection devices. Provided a wearable device is user-friendly, the technical skills required should not be a barrier for the current generation of people with Parkinson disease who are older or soon to be older.

One of the main findings of this study is that technology was perceived to be supplementary to appropriate health care, and it should not replace clinician involvement. In line with previous research [25], our participants prioritized information exchange between the patients and health care workers. Similarly, in another study [40], people with Parkinson disease saw the use of exergames in physiotherapy as being supplemental to physiotherapy sessions, not as their replacement.

Notwithstanding this, and in line with previous research [8], our study participants perceived that information from wearable devices could provide a better understanding of Parkinson disease and improve their symptoms. In addition, objective data may allow for more accurate management of Parkinson disease; specifically, symptom monitoring wearable devices could replace paper symptom diaries, which our participants cited as burdensome and limited. In line with studies by Ozanne et al [25] and AlMahadin et al [41], assessment methods are needed to increase the chance of effective treatment. As people with Parkinson disease experience symptom fluctuations as the day progresses, our participants, along with those in Ozanne et al [25], both identified the value of instant feedback. Prompt, real-time feedback on *switch on* or *switch off* states could help tailor their medication doses. Warnings about stress, lack of sleep, and falls would support their quality of life. Feedback from wearable devices could be tailored to individual preferences and used to trigger an intervention.

The survey demonstrated that people with Parkinson disease are more concerned with the functionality of a wearable device than its appearance. The focus group added a more nuanced viewpoint—the size and weight of the device were important but the esthetics were less significant. Other studies of people with Parkinson disease reported similar findings; usability, accuracy, unobtrusive design, and functionality were important aspects of device design [24,25]. However, in the research by Botros et al [22], participants did not feel fully at ease wearing sensors in public, which contrasts with the results in this study where participants reported no such issues. Nevertheless, participants did emphasize that wearable device design needs to consider Parkinson disease–related barriers such as poor hand motor function that may hinder donning and doffing and device interaction, a finding that was also illuminated in a previous wearable device trial by Fisher et al [24].

Our results demonstrated that special considerations should be given to Parkinson disease–related health barriers, cost of wearable devices, and user confidence with technology. The lack of motivation to use a wearable device should not be underestimated, as previous evidence suggests that user preference influences utility and sustained use [35]. Although people with Parkinson disease may have more medical needs than other older adults, both populations are more inclined to use a wearable device when they are motivated by the medical benefits of the device [42]. When a wearable device offers tangible improvements to their lives, older adults and people with Parkinson disease may be willing to sacrifice esthetic features in favor of useful device functions.

Many Parkinson disease–monitoring wearable devices exist [31]. Although none are all-encompassing, some devices accurately monitor certain Parkinson disease symptoms [12,43,44]. The symptoms of Parkinson disease manifest in various regions of the body, so it follows that the tools to measure these symptoms must be widely located. For example, gait speed is best measured near the center of mass, whereas dyskinesia is best measured by a wrist-worn device [31]. Our participants discussed the importance of ease of use, particularly for individuals lacking confidence in technology; consequently, use of multiple devices may be cumbersome for these individuals. Future designs should strive for a simple device that can measure multiple Parkinson disease symptoms.

In the timeline of device development, our study is a useful resource for the initial blueprint stage of wearable device design. Designers could search qualitative exploratory papers, such as ours, for inspiration and a framework to structure their design goals. User-centered design (UCD) is a broad philosophy that spans methodologies [45] and can help ensure wearable device usability, accessibility, affordability, and reliability, all of which can impact the quality of interaction of older adults with wearable devices [46]. The central tenet of UCD is end user participation [45]. A UCD approach is particularly beneficial when end users require a variety of features, such as for people with Parkinson disease. UCD, especially when health care is focused, can respond to people with Parkinson disease through an integrative and iterative development procedure focused on understanding the end users’ needs [47]. Many previous studies have used focus groups and surveys to evaluate existing devices as part of a UCD approach [48-50]. However, the participants in these studies were confined to discussions on predetermined devices. The variability of Parkinson disease severity, presentations, and symptoms means that a one-size-fits-all approach to device design is not appropriate for this population of patients. Living with Parkinson disease is a unique experience for each person, so consideration should be given to tailoring devices to individual needs, or at least designing a set of devices that can measure different symptoms.

The World Health Organization defines healthy aging as “the process of developing and maintaining the functional ability that enables wellbeing in older age” [51]. Individuals with chronic conditions, such as Parkinson disease, must manage a range of factors that contribute to their health. Self-management support acknowledges this and aids people in developing the knowledge, confidence, and skills they need to make optimal decisions about their health [52]. However, the health and social needs of people with Parkinson disease are often complex and change over time, with a wide range of functional abilities, where some individuals will maintain their independence and others will need help with their activities of daily living. Wearable devices will not cure Parkinson disease but they can add value to the user’s life by supporting them as they live with their disease. As defined by the World Health Organization, intrinsic capacity describes the composite of all the abilities of individuals and how those abilities develop over time [51]. This can be supported by wearable devices but each user’s intrinsic capacity will differ, and the wearable devices should also differ in their features. For example, if a wearable device can accurately track symptom fluctuations, the user may be able to fine-tune their medication regime and retain more of their intrinsic capacity. A device with multiple features will add value to users, ensuring a more effective person-centered device based on an individual’s intrinsic capacity. Considering intrinsic capacity may identify novel opportunities for disease management and has the potential to help wearable device designers better understand chronic conditions such as Parkinson disease and to design individualized technology to improve the health of their users.

Wearable devices that allow remote monitoring could improve health care access for rural patients or those unable to travel, resulting in a positive impact on health care outcomes and costs. Preceding the COVID-19 pandemic, the application of telehealth was more of an exception in health care, but the pandemic saw rapid implementation of remote health care across all disciplines [53]. This may continue in part after travel restrictions end, as health care professionals and patients become more familiar with and appreciate the advantages of telemedicine. Therefore, the future integration of technology with health care is crucial. As per Fasano et al [53], the COVID-19 pandemic is challenging the health care system to reflect on the modes of traditional access to care and to facilitate the remote management of people with Parkinson disease where needed to improve patient care. Remote care models, in which a person with Parkinson disease is not face-to-face in a clinical setting with their health care team, are enhanced by using wearable devices together with communication-based technologies such as videoconferencing [54]. Wearable devices allow remote monitoring of patient health data, which can then be fed to a database that can be accessed by the patient and their health care team [55]. These data may more accurately capture the symptoms of Parkinson disease in daily life, which may not be reflected in controlled clinical assessments. The pandemic motivated a notable shift to telemedicine within the Parkinson community and in a recent survey it was found that most respondents were satisfied with the experience, and a near majority expressed interest in continuing to use telemedicine after the COVID-19 outbreak had ended [56]. Although these technologies are not new, they are gaining greater application through the realization that telehealth and wearable monitoring can provide comparable and innovative levels of care [54].

### Limitations

Our study is one of a few that did not confine itself to focusing on gathering perspectives on a predetermined device and focused solely on a Parkinson disease population. It involved a mixed method approach to gain rich, in-depth data about the monitoring needs, values, and preferences of people with Parkinson disease. However, this study has several limitations. This is a small study of a geographically limited population, and the study design may have had a positive bias in attracting participants more familiar with technology. As participants were recruited from the same geographic region, it is possible that some individuals participated in both the survey and focus groups. The survey was anonymous and the response rate was unknown. An unspecified number of surveys were distributed by volunteers at PAI meetings and surveys were passively returned to the researchers. However, the survey participants represented a broad range of ages and were split between genders. Owing to their positive wording, the results from the Likert items must be interpreted with caution. They provide a slight indication that clinical usefulness is prioritized by people with Parkinson disease, but this result does not support a rigorous survey design. Our survey design was modified from Bergmann and McGregor [35]. However, an alternative study design, such as a discrete choice experiment, might better elicit participant preferences [57]. It could be argued that the sample was relatively homogenous as patients were recruited through a Parkinson disease support group, which may not be representative of all people with Parkinson disease. Moreover, because of the limited number of individuals involved, it was not possible to differentiate by ethnicity, educational level, background, and digital literacy. Future studies expanding on this work should aim to include people with Parkinson disease across a range of backgrounds and stages of Parkinson disease and recruit from a variety of settings.

### Conclusions

This study aimed to understand the views and needs of people with Parkinson disease regarding wearable devices for monitoring the disease and assisting in its management. People with Parkinson disease provided useful information about living with the disease, their current use of technology, and the desirable features of wearable devices, which designers and clinicians should consider. Although the participants were positive about wearable technology, they tended to see the use of wearable devices more for providing data for health care professionals than for providing feedback for themselves. They sacrifice esthetics for ease of use, function, and accuracy. Barriers to using wearable devices include poor hand function, average technology confidence, and potential costs. Considering intrinsic capacity can identify opportunities for disease management and has the potential to help wearable device designers better understand chronic conditions such as Parkinson disease, and in designing individualized technology to improve the health of its users. A UCD approach that includes people with Parkinson disease in the design of technology is likely to be rewarded with improved user engagement and adoption of wearable devices. This could result in better Parkinson disease symptoms and function data, leading to more accurate Parkinson disease management.

## Appendix

Multimedia Appendix 1 Survey including Bergmann mapping.

Multimedia Appendix 2 Interview schedule for focus group.

## Abbreviations

PAI: Parkinson’s Association of Ireland
SENDOC: Smart Sensor Devices for Rehabilitation and Connected Health
UCD: user-centered design

## References

[1] Postuma RB, Berg D, Stern M, Poewe W, Olanow CW, Oertel W, Obeso J, Marek K, Litvan I, Lang AE, Halliday G, Goetz CG, Gasser T, Dubois B, Chan P, Bloem BR, Adler CH, Deuschl G (2015). MDS clinical diagnostic criteria for Parkinson's disease. Mov Disord.

[2] Poewe W, Seppi K, Tanner CM, Halliday GM, Brundin P, Volkmann J, Schrag A, Lang AE (2017). Parkinson disease. Nat Rev Dis Primers.

[3] GBD 2016 Parkinson's Disease Collaborators (2018). Global, regional, and national burden of Parkinson's disease, 1990-2016: a systematic analysis for the Global Burden of Disease Study 2016. Lancet Neurol.

[4] Pringsheim T, Jette N, Frolkis A, Steeves TD (2014). The prevalence of Parkinson's disease: a systematic review and meta-analysis. Mov Disord.

[5] Kowal SL, Dall TM, Chakrabarti R, Storm MV, Jain A (2013). The current and projected economic burden of Parkinson's disease in the United States. Mov Disord.

[6] Post B, Merkus MP, de Bie RM, de Haan RJ, Speelman JD (2005). Unified Parkinson's disease rating scale motor examination: are ratings of nurses, residents in neurology, and movement disorders specialists interchangeable?. Mov Disord.

[7] Rovini E, Maremmani C, Cavallo F (2017). How wearable sensors can support Parkinson's disease diagnosis and treatment: a systematic review. Front Neurosci.

[8] Espay AJ, Bonato P, Nahab FB, Maetzler W, Dean JM, Klucken J, Eskofier BM, Merola A, Horak F, Lang AE, Reilmann R, Giuffrida J, Nieuwboer A, Horne M, Little MA, Litvan I, Simuni T, Dorsey ER, Burack MA, Kubota K, Kamondi A, Godinho C, Daneault J, Mitsi G, Krinke L, Hausdorff JM, Bloem BR, Papapetropoulos S, Movement Disorders Society Task Force on Technology (2016). Technology in Parkinson's disease: challenges and opportunities. Mov Disord.

[9] Memar S, Delrobaei M, Pieterman M, McIsaac K, Jog M (2018). Quantification of whole-body bradykinesia in Parkinson's disease participants using multiple inertial sensors. J Neurol Sci.

[10] López-Blanco R, Velasco MA, Méndez-Guerrero A, Romero JP, Del Castillo MD, Serrano JI, Rocon E, Benito-León J (2019). Smartwatch for the analysis of rest tremor in patients with Parkinson's disease. J Neurol Sci.

[11] Mancini M, Carlson-Kuhta P, Zampieri C, Nutt JG, Chiari L, Horak FB (2012). Postural sway as a marker of progression in Parkinson's disease: a pilot longitudinal study. Gait Posture.

[12] Rodríguez-Molinero A, Samà A, Pérez-Martínez DA, Pérez López C, Romagosa J, Bayés À, Sanz P, Calopa M, Gálvez-Barrón C, de Mingo E, Rodríguez Martín D, Gonzalo N, Formiga F, Cabestany J, Catalá A (2015). Validation of a portable device for mapping motor and gait disturbances in Parkinson's disease. JMIR Mhealth Uhealth.

[13] Ossig C, Antonini A, Buhmann C, Classen J, Csoti I, Falkenburger B, Schwarz M, Winkler J, Storch A (2016). Wearable sensor-based objective assessment of motor symptoms in Parkinson's disease. J Neural Transm (Vienna).

[14] Ginis P, Nieuwboer A, Dorfman M, Ferrari A, Gazit E, Canning CG, Rocchi L, Chiari L, Hausdorff JM, Mirelman A (2016). Feasibility and effects of home-based smartphone-delivered automated feedback training for gait in people with Parkinson's disease: a pilot randomized controlled trial. Parkinsonism Relat Disord.

[15] Maglione JE, Liu L, Neikrug AB, Poon T, Natarajan L, Calderon J, Avanzino JA, Corey-Bloom J, Palmer BW, Loredo JS, Ancoli-Israel S (2013). Actigraphy for the assessment of sleep measures in Parkinson's disease. Sleep.

[16] Ramdhani RA, Khojandi A, Shylo O, Kopell BH (2018). Optimizing clinical assessments in Parkinson's disease through the use of wearable sensors and data driven modeling. Front Comput Neurosci.

[17] Wendel N, Macpherson CE, Webber K, Hendron K, DeAngelis T, Colon-Semenza C, Ellis T (2018). Accuracy of activity trackers in Parkinson disease: should we prescribe them?. Phys Ther.

[18] Deane KH, Flaherty H, Daley DJ, Pascoe R, Penhale B, Clarke CE, Sackley C, Storey S (2014). Priority setting partnership to identify the top 10 research priorities for the management of Parkinson's disease. BMJ Open.

[19] Read J, Cable S, Löfqvist Charlotte, Iwarsson S, Bartl G, Schrag A (2019). Experiences of health services and unmet care needs of people with late-stage Parkinson's in England: A qualitative study. PLoS One.

[20] Schipper K, Dauwerse L, Hendrikx A, Leedekerken J, Abma T (2014). Living with Parkinson's disease: priorities for research suggested by patients. Parkinsonism Relat Disord.

[21] Serrano JA, Larsen F, Isaacs T, Matthews H, Duffen J, Riggare S, Capitanio F, Ferreira JJ, Domingos J, Maetzler W, Graessner H, SENSE-PARK Consortium (2015). Participatory design in Parkinson's research with focus on the symptomatic domains to be measured. J Parkinsons Dis.

[22] Botros A, Schütz N, Camenzind M, Urwyler P, Bolliger D, Vanbellingen T, Kistler R, Bohlhalter S, Müri RM, Mosimann UP, Nef T (2019). Long-term home-monitoring sensor technology in patients with Parkinson's disease-acceptance and adherence. Sensors (Basel).

[23] Cancela J, Pastorino M, Tzallas A, Tsipouras M, Rigas G, Arredondo M, Fotiadis D (2014). Wearability assessment of a wearable system for Parkinson's disease remote monitoring based on a body area network of sensors. Sensors (Basel).

[24] Fisher JM, Hammerla NY, Ploetz T, Andras P, Rochester L, Walker RW (2016). Unsupervised home monitoring of Parkinson's disease motor symptoms using body-worn accelerometers. Parkinsonism Relat Disord.

[25] Ozanne A, Johansson D, Hällgren Graneheim U, Malmgren K, Bergquist F, Alt Murphy M (2018). Wearables in epilepsy and Parkinson's disease-a focus group study. Acta Neurol Scand.

[26] Zhou S, Ogihara A, Nishimura S, Jin Q (2018). Analyzing the changes of health condition and social capital of elderly people using wearable devices. Health Inf Sci Syst.

[27] Kekade S, Hseieh C, Islam MM, Atique S, Mohammed Khalfan A, Li Y, Abdul SS (2018). The usefulness and actual use of wearable devices among the elderly population. Comput Methods Programs Biomed.

[28] Marston HR, Kroll M, Fink D, de Rosario H, Gschwind YJ (2016). Technology use, adoption and behavior in older adults: results from the iStoppFalls project. Educ Gerontol.

[29] Older adults and technology use. Pew Research Center.

[30] Zhao Y, Heida T, van Wegen EE, Bloem BR, van Wezel RJ (2015). E-health support in people with Parkinson's disease with smart glasses: a survey of user requirements and expectations in the Netherlands. J Parkinsons Dis.

[31] Sica M, Tedesco S, Crowe C, Kenny L, Moore K, Timmons S, Barton J, O'Flynn B, Komaris D (2021). Continuous home monitoring of Parkinson's disease using inertial sensors: a systematic review. PLoS One.

[32] Venkatesh V, Brown SA, Bala H (2013). Bridging the qualitative-quantitative divide: guidelines for conducting mixed methods research in information systems. MIS Q.

[33] (2011). Best practices for mixed methods research in the health sciences. National Institutes of Health.

[34] Atkins S, Lewin S, Smith H, Engel M, Fretheim A, Volmink J (2008). Conducting a meta-ethnography of qualitative literature: lessons learnt. BMC Med Res Methodol.

[35] Bergmann J, Chandaria V, McGregor A (2012). Wearable and implantable sensors: the patient's perspective. Sensors (Basel).

[36] Braun V, Clarke V (2006). Using thematic analysis in psychology. Qual Res Psychol.

[37] Creswell JW, Clark VL (2007). Designing and conducting mixed methods research. Aust N Z J Public Health.

[38] Fetters MD, Freshwater D (2015). Publishing a methodological mixed methods research article. J Mix Methods Res.

[39] Flick U, von Kardorff EV, Steinke I, Jenner B (2004). A Companion to Qualitative Research.

[40] Tobaigy A, Alshehri MA, Timmons S, Helal OF (2018). The feasibility of using exergames as a rehabilitation tool: the attitudes, awareness, opinions and experiences of physiotherapists, and older people towards exergames. J Phys Ther Sci.

[41] AlMahadin G, Lotfi A, Zysk E, Siena FL, Carthy MM, Breedon P (2020). Parkinson's disease: current assessment methods and wearable devices for evaluation of movement disorder motor symptoms - a patient and healthcare professional perspective. BMC Neurol.

[42] Moore K, O'Shea E, Kenny L, Barton J, Tedesco S, Sica M, Crowe C, Alamäki A, Condell J, Nordström A, Timmons S (2021). Older adults' experiences with using wearable devices: qualitative systematic review and meta-synthesis. JMIR Mhealth Uhealth.

[43] Rodríguez-Molinero A, Pérez-López C, Samà A, de Mingo E, Rodríguez-Martín D, Hernández-Vara J, Bayés À, Moral A, Álvarez R, Pérez-Martínez DA, Català A (2018). A kinematic sensor and algorithm to detect motor fluctuations in Parkinson disease: validation study under real conditions of use. JMIR Rehabil Assist Technol.

[44] Wang W, Lee J, Harrou F, Sun Y (2020). Early detection of Parkinson’s disease using deep learning and machine learning. IEEE Access.

[45] Abras C, Maloney-Krichmar D, Preece J (2004). User-centered design. Encyclopedia of Human-Computer Interaction.

[46] Lee C, Coughlin JF (2014). PERSPECTIVE: older adults' adoption of technology: an integrated approach to identifying determinants and barriers. J Prod Innov Manag.

[47] Czaja SJ, Boot WR, Charness N, Rogers WA (2019). Designing for Older Adults: Principles and Creative Human Factors Approaches, Third edition.

[48] Ehn M, Eriksson LC, Åkerberg N, Johansson A (2018). Activity monitors as support for older persons' physical activity in daily life: qualitative study of the users' experiences. JMIR Mhealth Uhealth.

[49] Demiris G, Chaudhuri S, Thompson HJ (2016). Older adults' experience with a novel fall detection device. Telemed J E Health.

[50] Thilo FJ, Hahn S, Halfens RJ, Schols JM (2019). Usability of a wearable fall detection prototype from the perspective of older people-A real field testing approach. J Clin Nurs.

[51] (2002). Active ageing a policy framework. World Health Organization.

[52] Grady PA, Gough LL (2014). Self-management: a comprehensive approach to management of chronic conditions. Am J Public Health.

[53] Fasano A, Antonini A, Katzenschlager R, Krack P, Odin P, Evans AH, Foltynie T, Volkmann J, Merello M (2020). Management of advanced therapies in Parkinson's disease patients in times of humanitarian crisis: the COVID-19 experience. Mov Disord Clin Pract.

[54] Mobbs RJ, Betteridge C (2020). WearTel: a potential solution to lack of objective patient assessment tools in remote care during the COVID-19 pandemic. J Spine Surg.

[55] Majumder S, Mondal T, Deen MJ (2017). Wearable sensors for remote health monitoring. Sensors (Basel).

[56] Feeney MP, Xu Y, Surface M, Shah H, Vanegas-Arroyave N, Chan AK, Delaney E, Przedborski S, Beck JC, Alcalay RN (2021). The impact of COVID-19 and social distancing on people with Parkinson's disease: a survey study. NPJ Parkinsons Dis.

[57] Louviere JJ, Flynn TN, Carson RT (2010). Discrete choice experiments are not conjoint analysis. J Choice Model.

