# Supplementary material for: The Views and Needs of People With Parkinson Disease Regarding Wearable Devices for Disease Monitoring: Mixed Methods Exploration
Source: JMIR Form Res. 2022 Jan 6;6(1):e27418. doi: 10.2196/27418 (PMC8778562; doi:10.2196/27418)
Supplement: Multimedia Appendix 1 [file formative_v6i1e27418_app1.docx]

Appendix 1

Survey

***Background Details***

1. Please indicate your age: _______
2. Are you: Male □ Female □

***Experience with technology***

1. Do you use any of the following technology?

Smart Phone □

Games Console (Wii, PlayStation, Xbox, Nintendo) □

Desktop Computer, Laptop, IPAD (tablet device) □

Other □

**Please state________________________________________**

1. How would you rate your own skills with technology (e.g. smartphone, desktop computer, games console)?

No skills **□**

Poor skills **□**

Average skills **□**

Good skills **□**

Excellent skills **□**

1. How frequently do you use technology (e.g. smartphone, desktop computer/tablet, games console)?

Everyday **□**

Most days **□**

Every week **□**

Rarely **□**

***Knowledge of Wearable Devices***

1. Had you heard of wearable smart devices/technology before this project?

Yes **□**  No **□**

1. Had you used a wearable technology device before the project? e.g. Fitbit Yes **□** No **□**
2. Do you currently use a store bought smart wearable device to monitor your health?

Yes **□**  No **□**

***Part b)*** Please read each of the following statements below and indicate your level of agreement (strongly disagree, disagree, neutral, agree and strongly agree) with each, by ticking one box which you feel most closely represents your opinion.

*[For visual clarity we have omitted our 5-point Likert scale and tick boxes, but instead present our survey statements mapped to the original Bergmann statements (which were rated by participants on a scale from 1 to 10)].*

| **Our Survey Statements** | **Original wording in Bergmann et.al 2012** | **Reason for Change** |
| --- | --- | --- |
| **Wearability** | | |
| 1. A *medical sensing device* should be comfortable to *wear.* | A body worn device should be comfortable. | Wording adjusted for clarity. |
| 2. A medical sensing device should be compact (light and small). | A body worn device should be compact (light and small). |  |
| 3. A medical sensing device should be discrete. | A body worn device should be discrete. |  |
| 4. A medical sensing device should be easy to attach to the body. | A body worn device should be easy to attach to the body. |  |
| 5. A medical sensing device should not affect your normal daily *routine.* | A body worn device should not affect normal daily behaviour. |  |
| 6. A medical sensing device should not detach *accidently.* | A body worn device should not detach from patient unless needed. | Wording adjusted for clarity. |
|  | A body worn device should blend in with different types of clothing to be worn with the device. | Felt to overlap with item 3. |
| **User Interface** | | |
| 7. A medical sensing device should be rechargeable*.* |  | Addition requested by people with Parkinson’s. |
| 8. A medical sensing device should be simple to operate (and maintain). | A body worn device should be simple to operate (and maintain). |  |
| 9. A medical sensing device should *be accompanied by* clear and readable instructions *for use.* | A body worn device should have clear and readable instructions. | Wording adjusted for clarity. |
|  | A body worn device should be recyclable | Statement omitted after feedback from co-researchers. |
|  | A body worn device should provide clear and useful results | Felt to overlap with other items. |
|  | A body worn device should minimize incorrect use of the system. | Felt to overlap with meaning of item 9 (clear instructions). |
| **Wearer Feedback** | | |
| 10. A medical sensing device should give instant feedback *to you.* | A body worn device should give instant feedback. | Wording adjusted for clarity. |
| 11. A medical sensing device should send alerts to the user. |  | Statement added after feedback from co-researchers, to distinguish between feedback and alerts. |
| 12. A medical sensing device should provide you with alerts of performance vs target (e.g. step count) | A body worn device should motivate the people to use it. | Wording amended to reflect motivation though performance alerts |
|  |  |  |
| **Clinical Accuracy** | | |
| 13. A medical sensing device should be reliable. | A body worn device should be reliable. |  |
| 14. A medical sensing device should increase the accuracy of current clinical *assessment.* | A body worn device should increase the accuracy of current clinical procedures. | Wording adjusted for clarity. |
| 15. A medical sensing device should reduce *your requirement to travel for clinical assessment.* | A body worn device should reduce travel to clinics and hospitals. | Wording adjusted for clarity. |
| 16. Results from your medical sensing device should form part of your clinical assessment. |  | Statement added after feedback from co-researchers. |
| 17. A medical sensing device would give you a sense of ownership of your own healthcare. |  | Addition requested by people with Parkinson’s |
| 18. A medical sensing device should work alongside your medical care team, instead of replacing them. |  | Addition requested by people with Parkinson’s |
|  | A body worn device should speed up currently used procedures. | Statement omitted as meaning unclear |
