# Supplementary material for: The Views and Needs of People With Parkinson Disease Regarding Wearable Devices for Disease Monitoring: Mixed Methods Exploration
Source: JMIR Form Res. 2022 Jan 6;6(1):e27418. doi: 10.2196/27418 (PMC8778562; doi:10.2196/27418)
Supplement: Multimedia Appendix 2 [file formative_v6i1e27418_app2.pdf]

Appendix 2

Interview Schedule for focus group

1. What is important to you in your day-to-day life?
2. What 'outcomes' should researchers focus on in studies with Parkinson's Disease?
3. What do you think of technology in general? Do you use it a lot?
4. What would be the benefits of technology for a person with Parkinson's Disease?
5. What do you think should be important design features? Participants were shown some examples of existing wearable devices (smart watch: which captures information about step count, distance, heart rate, calories burned, sleep, and glove prototype: which measures tremor, finger stiffness, and speed of movement).
6. Any potential barriers to technology?
